# Posterior sternoclavicular joint dislocation, a rare but “dangerous” injury

**DOI:** 10.11604/pamj.2021.39.81.29240

**Published:** 2021-05-27

**Authors:** Savas Deftereos, Georgios Drosos

**Affiliations:** 1 Radiology Department, Democritus University of Thrace, Alexandroupolis, Greece,; 2 Department of Orthopaedic Surgery, Democritus University of Thrace, Alexandroupolis, Greece

**Keywords:** Posterior sternoclavicular joint, dislocation, computed tomography

## Image in medicine

A 17 years of age male patient presented with pain and movement restriction of right upper-extremity after a fall from his bicycle. On examination local swelling, tenderness, and depression of the medial end of the clavicle were present. Any attempt of shoulder movement was causing excruciating pain in this area. There was no other apparent injury and apart from the above-mentioned symptoms and clinical findings, all patients´ vital signs were normal. No prominent fracture or other pathology were noticed on X-rays. The clinical findings were compatible with an injury of the sternoclavicular joint; a posterior sternoclavicular joint (SCJ) dislocation in particular. Posterior dislocation of the SCJ can be associated with injuries of the structures that lie behind the manubrium resulting in life threatening complications such as neurovascular, tracheal and oesophageal injuries. Therefor prompt diagnosis and treatment is required. Based on the clinical findings, a computed tomography (CT) was performed and the clinical suspicion was confirmed (A,B). The dislocation was reduced under general anesthesia by closed means, followed by arm immobilization.

**Figure 1:**
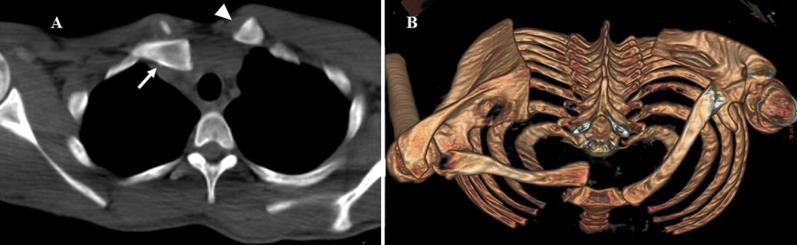
computerized tomography (CT) scan depicting axial (A) views of a right posterior sternoclavicular joint dislocation (arrow) and normal positioned left sternoclavicular joint (arrowhead) and 3D reconstruction (B) (notice the narrowed space behind manubrium on the 3D-reconstruction image)

